# Quantitative testing of the methodology for genome size estimation in plants using flow cytometry: a case study of the *Primulina* genus

**DOI:** 10.3389/fpls.2015.00354

**Published:** 2015-05-19

**Authors:** Jing Wang, Juan Liu, Ming Kang

**Affiliations:** Key Laboratory of Plant Resources Conservation and Sustainable Utilization, South China Botanical Garden, Chinese Academy of SciencesGuangzhou, China

**Keywords:** flow cytometry, genome size, Gesneriaceae, methodology, *Primulina*

## Abstract

Flow cytometry (FCM) is a commonly used method for estimating genome size in many organisms. The use of FCM in plants is influenced by endogenous fluorescence inhibitors and may cause an inaccurate estimation of genome size; thus, falsifying the relationship between genome size and phenotypic traits/ecological performance. Quantitative optimization of FCM methodology minimizes such errors, yet there are few studies detailing this methodology. We selected the genus *Primulina*, one of the most representative and diverse genera of the Old World Gesneriaceae, to evaluate the methodology effect on determining genome size. Our results showed that buffer choice significantly affected genome size estimation in six out of the eight species examined and altered the 2C-value (DNA content) by as much as 21.4%. The staining duration and propidium iodide (PI) concentration slightly affected the 2C-value. Our experiments showed better histogram quality when the samples were stained for 40 min at a PI concentration of 100 μg ml^−1^. The quality of the estimates was not improved by 1-day incubation in the dark at 4°C or by centrifugation. Thus, our study determined an optimum protocol for genome size measurement in *Primulina*: LB01 buffer supplemented with 100 μg ml^−1^ PI and stained for 40 min. This protocol also demonstrated a high universality in other Gesneriaceae genera. We report the genome size of nine Gesneriaceae species for the first time. The results showed substantial genome size variation both within and among the species, with the 2C-value ranging between 1.62 and 2.71 pg. Our study highlights the necessity of optimizing the FCM methodology prior to obtaining reliable genome size estimates in a given taxon.

## Introduction

Genome size (C-value or the haploid nuclear DNA content) is significantly correlated with cell/nucleus sizes, cellular processes (e.g., the DNA synthesis rate), and a range of ecological characteristics (Beaulieu et al., 2008; Greilhuber and Leitch, 2013). Genome size has fundamental biological importance with considerable applications in a wide range of fields, including ecology, cell and molecular biology, systematics, and evolution (Bennett and Leitch, 2005, 2011; Veselý et al., 2012). Genome size varies a remarkable 2400-fold across the angiosperm flora (Pellicer et al., 2010) and even exhibits substantial variation among closely related species (Duchoslav et al., 2013; Fleischmann et al., 2014). It is an important biological parameter that has been increasingly used to clarify evolutionary patterns and adaptation mechanisms of plants (e.g., Huang et al., 2013; Kang et al., 2014; Jordan et al., 2015). Rapid advancements in whole-genome sequencing of non-model plants further highlight the importance of accurately estimating genome size.

Flow cytometry (FCM) has been considered a fast, sensitive technique for determining the genome size of plants since Galbraith et al. (1983) developed a rapid and simple method for isolation of nuclei by chopping leaf tissues in a lysis buffer. However, the accuracy of FCM results in plants has been reportedly influenced by secondary metabolites, such as tannic acid (Loureiro et al., 2006), anthocyanins (Bennett et al., 2008), and extremely mucilaginous compounds (Cires et al., 2011). The detrimental impact of endogenous fluorescence inhibitors on genome size estimation has led researchers to see k effective methods to prevent or minimize such effects (e.g., Loureiro et al., 2006, 2007a; Bennett et al., 2008). It was reported that simultaneous processing of both target and standard samples followed by a comparison of the data based on internal and external standards can effectively ameliorate the secondary metabolites' influence on genome size estimations (Price et al., 2000; Noirot et al., 2005). Other methods include selecting suitable nuclei isolation buffers, using antioxidant compounds (Doležel and Bartoš, 2005; Loureiro et al., 2006), determining the optimum stain concentration and staining duration (Loureiro et al., 2006; Doležel et al., 2007), and centrifuging nuclear suspensions and discarding the supernatant (Doležel and Bartoš, 2005). To reduce experimental errors, all of the samples, including both target and standard plants, should be maintained under the same environmental conditions (Noirot et al., 2005) and distinct replicates should be performed (Doležel and Bartoš, 2005; Bennett et al., 2008). On the other hand, recent advances in FCM methods have enabled rigorous documentation of inter- and intra-specific genome size variation (Bainard et al., 2010; Cires et al., 2011). However, only a few studies have evaluated the effect of methodology on genome size estimations in a given taxon (Bainard et al., 2010; Cires et al., 2011).

Genome sizes have been estimated for more than 7500 angiosperm species, belonging to more than 50% of the angiosperm families and representing approximately 2% of flowering plant species (APG III, 2009; Bennett and Leitch, 2012). However, these studies are confined to particular regions (e.g., Europe and North America), whereas plant genome size in other areas with higher species richness and endemism remains poorly understood (Bennett and Leitch, 2011). The Southern and Southwestern parts of China boast of 20,000 plant species and are considered the most endemic-rich subtropical flora areas of the world. We have initiated a large-scale project to examine the genome size in the flora of Southern China. The largest genus of the old world Gesneriaceae, *Primulina* (Wang et al., 2011; Weber et al., 2011), is a monophyletic group with more than 150 perennial species that is widely distributed throughout the karst regions of China and adjacent countries in Southeast Asia. Approximately 120 *Primulina* species (85%) are endemic to Southern and Southwestern China. The high microhabitat specialization and species richness of the genus makes *Primulina* an ideal non-model system for studying speciation and adaptation. Moreover, an accurate estimation of genome size in *Primulina* is important to understand the pattern of genome size variation and its role in evolutionary adaptation and speciation mechanism.

The abundant and diverse secondary metabolites detected in Gesneriaceae (Verdan and Stefanello, 2012) make it critical to evaluate the effect of methodologies and determine the optimal FCM protocol prior to estimating genome size of *Primulina* species. The present study was conducted to quantify the impact of different FCM methodologies on genome size measurement in *Primulina*. These include evaluating the performance of different nuclei extraction buffers, staining regimens, dark and cold conditions, and centrifugation during sample preparation. We quantitatively optimized a FCM methodology suitable for *Primulina* and validated the protocol's universality in several other genera of Gesneriaceae. Additionally, in the present study, we report the genome size of nine species of the Gesneriaceae family in China.

## Materials and methods

### Plant material

Eight *Primulina* species (*P. linearifolia, P. huaijiensis, P. heterotricha, P. liguliformis, P. roseoalba, P. lunglinensis, P. hedyotidea*, and *P. subrhomboidea*) with different phenotypic and ecological traits were selected to test the effects of FCM methodology. Because our preliminary survey revealed the 2C-value of the eight species ranged between 1.0 and 2.1 pg, *Solanum lycopersicum* cv. “Stupické polni rané” (2C = 1.96 pg, Doležel et al., 1992) was selected as an appropriate primary reference standard. *Oryza sativa* ssp. *japonica* (2C = 0.86–0.90 pg, Arumuganathan and Earle, 1991), whose 2C-value was further calibrated against *S. lycopersicum* (10 replicates on different days), was chosen as a secondary reference standard. The seeds of *O. sativa* ssp. *japonica* and *S. lycopersicum* were obtained from the South China Botanical Garden, the Chinese Academy of Sciences, and the Laboratory of Molecular Cytogenetics and Cytometry (Olomouc, Czech Republic), respectively. Seedlings of the references were grown and kept in greenhouses at the South China Botanical Garden.

### Sample preparation

We conducted preliminary analyses to determine the appropriate amount of sample required to produce sufficient nuclei counts and good-quality histograms. Approximately 20 mg of fresh tissue from the young leaves of samples and standards were used, respectively. Briefly, the sample and standard tissues were co-chopped in 1 ml of cold buffer on ice. A razor blade with a single edge was used for chopping each sample in a Petri dish as described by Galbraith et al. (1983). Samples were chopped quickly (approximately 45 s) but not intensely to minimize the release of cytosolic compounds. A 50-μm mesh filter was used to filter the resulting homogenate. RNase A (Sigma, Cream Ridge, USA) was added at a final concentration of 50 μg ml^−1^, PI (Sigma) was used according to the methodology outlined below, and samples were incubated in the dark and on ice.

### Experimental design

#### Testing the presence of endogenous inhibitors

The Partec CyStain PI Absolute P kit (Partec GmbH, Münster, Germany) was used to test for a reduction in PI fluorescence of the reference standard by secondary metabolites in *Primulina*, given that it has been used in Gesneriaceae (Zaitlin and Pierce, 2010). Leaves of standard (*S. lycopersicum* or *O. sativa* ssp. *japonica*) were independently chopped and simultaneously processed (co-chopped) with leaves of *Primulina*. After staining, mean PI fluorescence was measured for standards independently and simultaneously processed, respectively. Three replicates were completed.

#### Buffer screening

The effect of nuclei isolation buffer on genome size determination was tested for eight buffers across all eight species. In addition to the Partec CyStain PI Absolute P kit, seven buffers were selected from the 10 most-commonly used non-commercial FCM buffers Loureiro et al., 2007a,b. These buffers were de Laat's (de Laat and Blaas, 1984; modified as in Kron and Husband, 2009), Galbraith's (Galbraith et al., 1983), General Purpose (Loureiro et al., 2007a), LB01 (Doležel et al., 1989), MgSO_4_ (Arumuganathan and Earle, 1991), Tris-MgCl_2_ (Pfosser et al., 1995), and Woody Plant (Loureiro et al., 2007a). Four replicates of each species were analyzed with each buffer. PI was used at a concentration of 50 μg ml^−1^ for 20 min, according to protocol described by Price et al. (2000).

#### Determination of staining duration

Samples of *P. linearifolia, P. huaijiensis, P. heterotricha*, and *P. liguliformis* were randomly selected from the eight species to investigate the effect of staining duration. Buffer LB01 was chosen due to its relatively high-resolution performance during the buffer test. Each sample was examined using the following time course: 5, 10, 15, 20, 40, 60, and 120 min. Because each sample was analyzed seven times, we increased the tissue amount to 40 mg for samples and standards and the volume of isolation buffer to 2 ml. PI was used at 50 μg ml^−1^ and samples were incubated on ice between runs. The experiment was repeated three times for each species.

#### Determination of stain concentration

According to the results from the tests described above, the optimal conditions included LB01 buffer and staining duration of 40 min were then tested for any effect of stain concentration. The same four species as previously described were used. The PI concentrations examined included: 10, 25, 50, 100, 150, and 200 μg ml^−1^. Similarly, a larger volume of homogenate (2 ml) was needed to perform each analysis at the six different PI concentrations. Four replicates were analyzed for each species at each stain concentration.

#### Effect of darkness and centrifugation

The samples of the same four species were analyzed after incubation in the dark at 4°C for 24 h to check the possible effect of darkness on the quality of histograms. Alternatively, the homogenates were centrifuged at 500 × g for 10 min at 4°C to test the effect of centrifugation, according to the procedure used in *Sinningia* (Gesneriaceae) (Zaitlin and Pierce, 2010). After centrifugation, the supernatant was carefully decanted, and the pellet was gently re-suspended in 850 μl of new extraction buffer. Three replicates were tested for each species.

### Flow cytometric analyses

Analysis of stained samples was performed on a Partec CyFlow Space (Partec, Münster, Germany) equipped with a 20-mW sapphire laser, a 25-mW solid-state laser, and a 50-mW UV-LED operating at 488, 638, and 365 nm, respectively, and the fluorescence intensity of 10,000 particles was recorded. Before each use, we calibrated the instrument using 3-μm calibration beads (Partec, Münster, Germany). The forward scatter (FSC), side scatter (SSC) and orange fluorescence (FL2: 590 nm ± 25) were measured for each sample. These parameters were visualized alone and in combined histograms as follows: FL2 vs. FSC, FL2 vs. SSC, and FSC vs. SSC.

**Figure 1:**
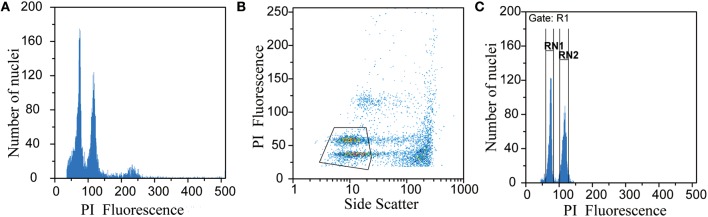
**An example of the gating procedure using data from *Primulina linearifolia***. Ungated histogram **(A)**, scatterplot with manually drawn polygon gate **(B)**, histogram with polygon gate applied to remove debris and regions drawn to determine peak location and CV **(C)**, gating procedure completed with FloMax Software (Partec Version 2.80, 2012).

The nuclei number and coefficient of variation (CV) were obtained for each peak of interest (sample and standard) using the gating function in the FloMax Software by Partec (Version 2.80, 2012). Polygon gates were drawn manually around regions of interest on the scattergram of PI fluorescence vs. side scatter to remove the interference of debris particles (Figure 1). To determine the fluorescence and CV of each peak, regions were developed around the histograms of interest (Figure 1). Although most of the genome size literatures recommend CV be set below 5% over 5000 or 10,000 nuclei analyzed, it was expected that some of the methodology used here would not produce optimal results. For each histogram, the nuclei count and CV for each peak of interest were used to calculate relative standard error (RSE) using the formula RSE = SE/mean, which can be calculated as peak CV%/ v(peak nuclei count), as described by Bainard et al. (2010). The ability to consider both the number of nuclei measured as well as the CV is especially helpful when data might otherwise be ignored due to low nuclei counts or high CV-values.

The nuclear DNA content of *Primulina* was determined according to the following formula: Standard 2C-value (pg) = (Sample peak mean/Standard peak mean) ^*^ nuclear DNA content of the reference Standard (pg).

### Statistical analyses

Analysis of the buffer effect data was performed using a mixed model analysis of variance (ANOVA) with the buffer as the fixed variable and the data as the random variable. A mixed model ANOVA was used to identify the effect of the staining period, with time as fixed repeated variable. To analyze the stain concentration data, a general linear model was used with concentration as the fixed variable. Tukey's HSD *post hoc* test (*P* = 0.05) was added to evaluate the significance of the differences in means within each test and for each species. Model residuals were used to determine whether the underlying assumptions of homogeneity of variance and normality were met. Data analysis was performed using SPSS Version 3.1 (SPSS Inc., Chicago, IL, USA).

### Testing the optimized methodology in other genera

After optimizing, we further tested the optimized methodology's universality in Gesneriaceae in South China Karst. We examined 32 populations from nine species that belong to four other genera, including *Hemiboea, Lysionotus, Briggsia*, and *Aeschynanthus*. Herbarium vouchers for these species have been deposited in the IBSC (South China Botanical Garden, CAS).

## Results

### Compounds in *Primulina* leaves inhibit PI-fluorescence

**Figure 2:**
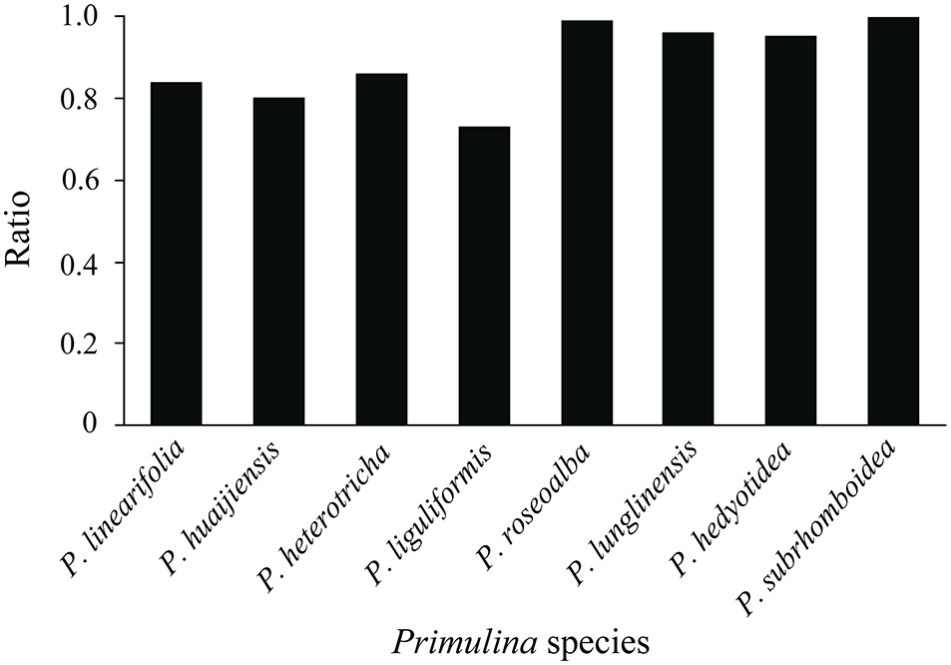
**The ratio of the mean fluorescence of nuclei (Standard 1) from simultaneously processed standard (*Solanum lycopersicum* or *Oryza sativa* ssp. *japonica*) with *Primulina* leaves to the mean fluorescence of nuclei (Standard 2) from an independently processed standard using the Partec CyStain PI Absolute P kit**.

The inhibition tests indicated the ratio of the mean fluorescence of nuclei (Standard 1) from simultaneously processed standard (*S. lycopersicum* or *O. sativa* ssp. *japonica*) with *Primulina* leaves to the mean fluorescence of nuclei (Standard 2) from independently processed standards. These ratios ranged between 0.73 and 1.00 (Table S1, Figure 2). The reduced fluorescence of nuclei from simultaneously processed standards compared to the nuclei from independently processed standard leaves suggests the presence of endogenous inhibitors in *Primulina*. The fluorescence reduction occurred in seven out of eight *Primulina* species, with the exception being *P. subrhomboidea*. Moreover, the effects of inhibition differ among species, which is more apparent in *P. liguliformis, P. huaijiensis, P. linearifolia*, and *P. heterotricha*.

### Effect of various buffers on genome size estimation

The buffers tested had a significant effect on the 2C-value in six out of the eight species analyzed (*P* < 0.05), with the exceptions being *P. hedyotidea* and *P. subrhomboidea* (Table 1). Although the absolute differences in DNA content were small (0.120–0.302 pg/2C), the largest percent differences ([maximum estimate - minimum estimate]/mean) were substantial. The values varied by 14.6% for *P. linearifolia* (MgSO_4_ and Galbraith's), 21.4% for *P. huaijiensis* (de Laat's and Partec), 15.9% for *P. heterotricha* (General purpose and de Laat's), 19.8% for *P. liguliformis* (Woody plant and General purpose), 14.9% for *P. roseoalba* (Woody plant and Tris-MgCl_2_), 9.2% for *P. lunglinensis* (Tris-MgCl_2_ and Galbraith's), 7.7% for *P. hedyotidea* (Galbraith's and MgSO_4_), and 6.83% for *P. subrhomboidea* (Woody plant buffer and MgSO_4_). The estimate patterns generated by various buffers was different across species, although both Partec and Woody plant buffer usually obtained estimates above the mean and MgSO_4_ and Galbraith's buffer tended to produce estimates below the mean. Buffers de Laat's, LB01, Tris-MgCl_2_ and General purpose produced estimates relatively close to the mean values across the analyzed species.

Table 1**Effect of various buffers on the 2C-value estimates of eight *Primulina* species**.

**Table:** 

**Buffer**	***P. linearifolia***			***P. huaijiensis***			***P. heterotricha***		
	**Mean 2C-value (pg) ± SD**	**Mean RSE (%) sample**	**Mean RSE (%) standard**	**Mean 2C-value (pg) ± SD**	**Mean RSE (%) sample**	**Mean RSE (%) standard**	**Mean 2C-value (pg) ± SD**	**Mean RSE (%) sample**	**Mean RSE (%) standard**
MgSO_4_	1.234 ± 0.008a	0.087	0.061	1.113 ± 0.008a	0.120	0.071	1.398 ± 0.036a	0.062	0.100
Partec	1.140 ± 0.018b	0.112	0.069	0.992 ± 0.013b	0.252	0.164	1.328 ± 0.038ab	0.126	0.140
de Laat's	1.188 ± 0.007c	0.109	0.089	1.224 ± 0.012c	0.155	0.126	1.236 ± 0.031c	0.122	0.126
Galbraith's	1.064 ± 0.022d	0.247	0.122	1.021 ± 0.011b	0.140	0.104	1.252 ± 0.047bc	0.102	0.124
LB01	1.190 ± 0.006ac	0.071	0.057	1.095 ± 0.005ad	0.094	0.066	1.414 ± 0.007a	0.072	0.107
Tris-MgCl_2_	1.132 ± 0.014b	0.184	0.080	1.086 ± 0.008ad	0.002	0.100	1.275 ± 0.059bc	0.092	0.150
General purpose	1.140 ± 0.008b	0.118	0.066	1.128 ± 0.044a	0.134	0.102	1.448 ± 0.015ad	0.086	0.159
Woody plant	1.192 ± 0.009c	0.096	0.004	1.068 ± 0.09d	0.234	0.155	1.340 ± 0.030ab	0.120	0.215
Mean	1.162 ± 0.014			1.086 ± 0.014			1.336 ± 0.032

**Table:** 

**Buffer**	***P. linearifolia***			***P. huaijiensis***			***P. heterotricha***		
	**Mean 2C-value (pg) ± SD**	**Mean RSE (%) sample**	**Mean RSE (%) standard**	**Mean 2C-value (pg) ± SD**	**Mean RSE (%) sample**	**Mean RSE (%) standard**	**Mean 2C-value (pg) ± SD**	**Mean RSE (%) sample**	**Mean RSE (%) standard**
MgSO_4_	1.435 ± 0.010ac	0.071	0.092	1.996 ± 0.032a	0.080	0.102	1.972 ± 0.022a	0.084	0.095
Partec	1.668 ± 0.050b	0.091	0.123	2.000 ± 0.041a	0.109	0.147	2.136 ± 0.052b	0.088	0.131
de Laat's	1.452 ± 0.015ac	0.060	0.142	2.012 ± 0.032a	0.095	0.180	2.010 ± 0.017abc	0.089	0.110
Galbraith's	1.408 ± 0.047ac	0.116	0.160	1.934 ± 0.033a	0.110	0.126	1.951 ± 0.019ac	0.118	0.140
LB01	1.434 ± 0.008ac	0.071	0.104	2.021 ± 0.027a	0.071	0.112	2.014 ± 0.016ab	0.090	0.107
Tris-MgCl_2_	1.463 ± 0.025ac	0.082	0.147	1.774 ± 0.054b	0.098	0.113	2.046 ± 0.128ab	0.118	0.184
General purpose	1.384 ± 0.017ad	0.093	0.119	2.044 ± 0.142a	0.085	0.149	2.006 ± 0.049ab	0.080	0.102
Woody Plant	1.468 ± 0.014ac	0.067	0.143	2.071 ± 0.055a	0.100	0.203	2.030 ± 0.066ab	0.061	0.220
Mean	1.436 ± 0.024			1.984 ± 0.056			2.020 ± 0.048

**Table:** 

**Buffer**	***P. linearifolia***			***P. huaijiensis***		
	**Mean 2C-value (pg) ± SD**	**Mean RSE (%) sample**	**Mean RSE (%) standard**	**Mean 2C-value (pg) ± SD**	**Mean RSE (%) sample**	**Mean RSE (%) standard**
MgSO_4_	1.516 ± 0.040a	0.084	0.098	1.818 ± 0.027a	0.070	0.080
Partec	1.589 ± 0.084a	0.161	0.164	1.883 ± 0.041a	0.094	0.099
de Laat's	1.548 ± 0.073a	0.147	0.196	1.822 ± 0.042a	0.088	0.105
Galbraith's	1.636 ± 0.131a	0.144	0.108	1.882 ± 0.123a	0.096	0.142
LB01	1.548 ± 0.006a	0.081	0.126	1.853 ± 0.016a	0.072	0.098
Tris-MgCl_2_	1.596 ± 0.072a	0.187	0.156	1.896 ± 0.048a	0.072	0.115
General purpose	1.557 ± 0.131a	0.122	0.109	1.889 ± 0.057a	0.062	0.113
Woody Plant	1.560 ± 0.055a	0.186	0.135	1.946 ± 0.018a	0.053	0.146
Mean	1.568 ± 0.074			1.874 ± 0.048		

*Letters shared (within each species) indicated no significant differences in mean 2C-value (Tukey's HSD test P = 0.05). SD, Standard deviation. Each value is the mean of four replicates*.

**Figure 3:**
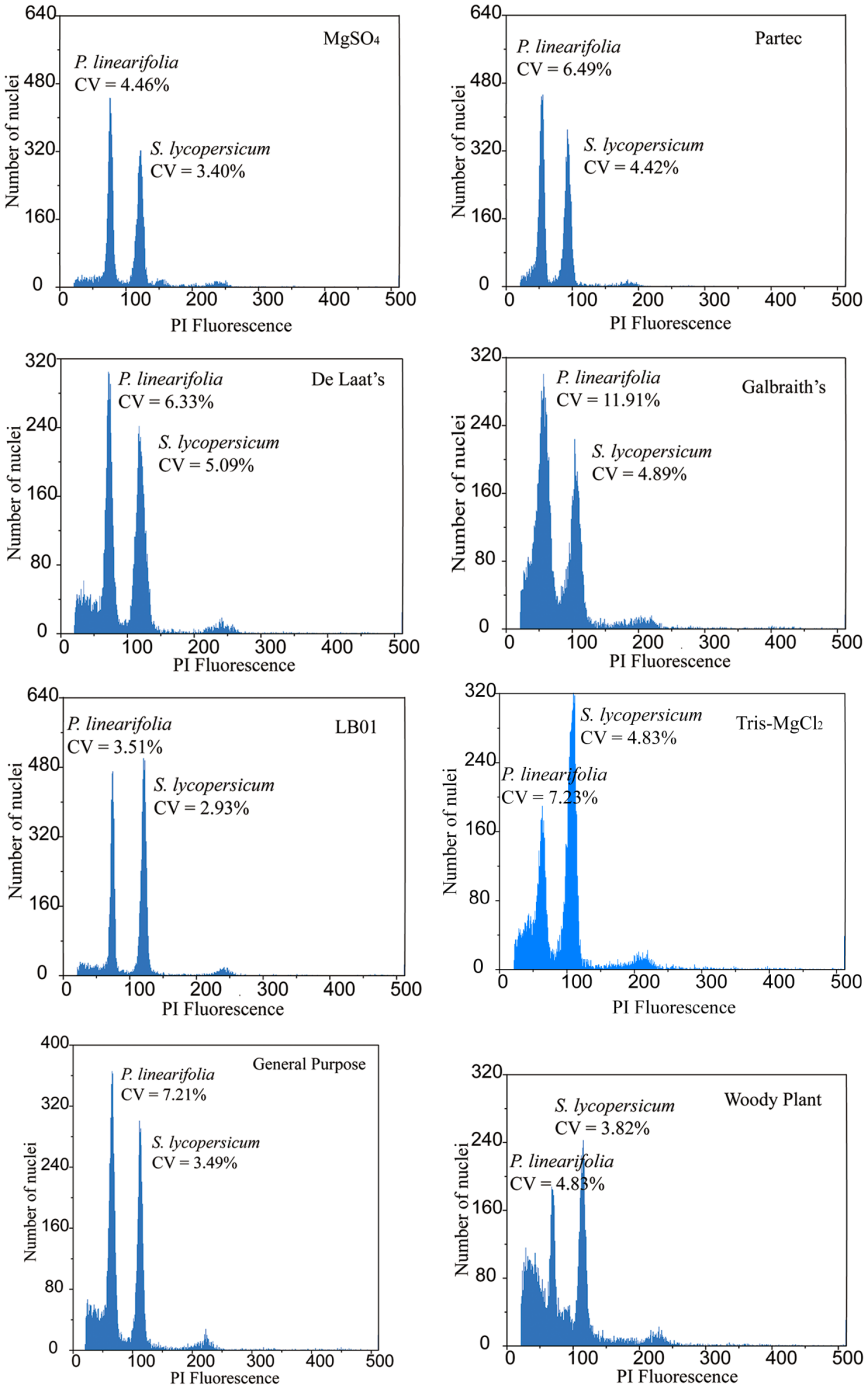
**Ungated histograms showing the range of quality of *Primulina linearifolia* obtained with eight different buffers**.

The buffers used also produced variation in resolution for each of the eight species (Table 1, Figure 3). We used the SE of the mean 2C-value and RSE to measure quality. A small mean SE-value indicated high consistency within the 2C-value estimated over replicates, whereas the mean RSE reflected the quality of histograms (combining the CV-value and nuclei count obtained for each peak). MgSO_4_, de Laat's and LB01 buffers produced low mean SE estimates across species. Low CV and RSEs were consistently produced from MgSO_4_ and LB01 buffer (with sample RSE varying between 0.062 and 0.120% across species) (Table 1, Table S2). Woody plant buffer showed low RSE-values for most species, with the exception for *P. hedyotidea* (0.186%) and *P. huaijiensis* (0.234%). Partec, de Laat's, Galbraith's, and Tris-MgCl_2_ buffer produced relatively high RSE-values for the eight species (sample RSE ranging from 0.002 to 0.247%). Such effects on resolution of different buffers were also demonstrated by the scatterplots of SSC vs. PI fluorescence and FSC vs. PI fluorescence. Significantly larger amounts of debris were usually obtained from the buffers that produced poor-quality histograms. Histograms with less compact clusters of nuclei tended to appear with buffers that generated high RSEs, indicating that the buffer used affected the nuclei characteristics, such as relative size (FSC), relative surface complexity estimate (SSC), and the fluorescence (Figure S1).

### Effect of staining regimen on genome size estimation

Our results showed that staining duration affected the relative fluorescence of both the sample and the standard but had a negligible effect on the 2C-value estimates. The 2C-value estimates of the analyzed taxa showed a maximum change of only 0.096 pg over 120 min, and the differences between estimates were not significant (Table 2). Histogram quality was better (lower RSE) at the 30 and 40 min staining periods than other periods, for all species. Stain concentration significantly affected the genome size estimate for *P. linearifolia* (*P* < 0.05) but not for *P. huaijiensis, P. heterotricha* or *P. liguliformis* (Table 3). The genome size estimates were generally low across species at PI concentrations of 10 and 25 μg ml^−1^. The largest difference in the 2C-value estimates for each species was 6.7% for *P. linearifolia*, 1.0% for *P. huaijiensis*, 4.5% for *P. heterotricha*, and 5.9% for *P. liguliformis*. The histogram quality varied with different staining concentrations. Generally, the moderate concentration of PI (100 μg ml^−1^) generated the best quality histograms (lowest RSE).

**Table 2:** **The effect of propidium iodide (PI) staining duration on the 2C-value estimates of four *Primulina* species**.

**Staning during (min)**	***P. linearifolia***					***P. huaijiensis***				
	**Mean PI fluorescence**		**Mean 2C-value (pg) ± SD**	**Mean RSE (%) sample**	**Mean RSE (%) standard**	**Mean PI fluorescence**		**Mean 2C-value (pg) ± SD**	**Mean RSE (%) sample**	**Mean RSE (%) standard**
	**Sample**	**Standard**				**Sample**	**Standard**			
5	71.36	114.13	1.226 ± 0.016a	0.295	0.191	65.51	116.56	1.130 ± 0.022a	0.274	0.100
10	73.92	117.38	1.234 ± 0.016a	0.224	0.166	66.00	116.26	1.140 ± 0.024a	0.298	0.117
20	75.0	118.63	1.218 ± 0.028a	0.274	0.196	64.14	112.25	1.150 ± 0.024a	0.273	0.092
30	75.43	119.31	1.224 ± 0.018a	0.240	0.174	64.26	114.02	1.112 ± 0.022a	0.246	0.089
40	74.90	117.68	1.246 ± 0.010a	0.170	0.136	66.72	115.42	1.162 ± 0.024a	0.243	0.102
50	73.90	116.86	1.234 ± 0.012a	0.202	0.141	62.44	111.59	1.106 ± 0.022a	0.266	0.100
60	73.04	116.57	1.218 ± 0.020a	0.181	0.143	63.27	112.57	1.102 ± 0.022a	0.317	0.118
120	73.92	117.38	1.240 ± 0.002a	0.207	0.154	58.12	105.4	1.084 ± 0.022a	0.361	0.127
Mean	73.94	117.24	1.230 ± 0.016			63.81	113.01	1.124 ± 0.022		
**Staning during (min)**	***P. heterotricha***					***P. liguliformis***				
	**Mean PI fluorescence**		**Mean 2C-value (pg) ± SD**	**Mean RSE (%) sample**	**Mean RSE (%) standard**	**Mean PI fluorescence**		**Mean 2C-value (pg) ± SD**	**Mean RSE (%) sample**	**Mean RSE (%) standard**
	**Sample**	**Standard**				**Sample**	**Standard**			
5	82.54	54.11	1.376 ± 0.032a	0.172	0.181	90.28	53.09	1.534 ± 0.034a	0.190	0.248
10	82.49	54.00	1.378 ± 0.012a	0.182	0.204	90.73	52.98	1.558 ± 0.036a	0.125	0.192
20	79.29	52.33	1.368 ± 0.006a	0.163	0.173	88.44	51.24	1.556 ± 0.036a	0.161	0.244
30	82.11	54.83	1.352 ± 0.040a	0.171	0.187	85.68	49.58	1.560 ± 0.046a	0.146	0.237
40	81.18	53.81	1.362 ± 0.030a	0.162	0.166	84.61	48.23	1.582 ± 0.024a	0.139	0.232
50	76.95	50.32	1.382 ± 0.036a	0.196	0.244	85.58	49.68	1.558 ± 0.066a	0.136	0.209
60	78.50	51.27	1.382 ± 0.020a	0.179	0.199	84.26	49.43	1.542 ± 0.066a	0.175	0.280
120	84.47	57.86	1.324 ± 0.060a	0.170	0.203	84.28	51.15	1.486 ± 0.016a	0.146	0.232
Mean	80.94	53.57	1.366 ± 0.030			86.73	50.67	1.548 ± 0.040		

*Letters shared (within each species) indicated no significant differences in mean 2C-value (Tukey's HSD test P = 0.05). SD, Standard deviation. Each value is the mean of three replicates*.

**Table 3:** **The effect of propidium iodide (PI) concentration on the 2C-value estimates of four *Primulina* species**.

**PI Conc. (μg/ml)**	***P. linearifolia***					***P. huaijiensis***				
	**Mean PI fluorescence**		**Mean 2C-value (pg) ± SD**	**Mean RSE (%) sample**	**Mean RSE (%) standard**	**Mean PI fluorescence**		**Mean 2C-value (pg) ± SD**	**Mean RSE (%) sample**	**Mean RSE (%) standard**
	**Sample**	**Standard**				**Sample**	**Standard**			
10	48.50	81.73	1.104 ± 0.048a	0.245	0.153	49.98	89.57	1.106 ± 0.030a	0.169	0.123
25	51.05	91.74	1.140 ± 0.018ac	0.172	0.110	54.11	96.90	1.106 ± 0.034a	0.156	0.112
50	57.85	97.86	1.158 ± 0.020bc	0.178	0.118	58.62	104.36	1.112 ± 0.030a	0.152	0.117
100	62.89	105.65	1.166 ± 0.002bc	0.150	0.106	63.04	111.38	1.120 ± 0.02a	0.147	0.117
150	65.60	109.99	1.170 ± 0.002bc	0.152	0.102	65.74	115.70	1.126 ± 0.028a	0.147	0.117
200	68.76	114.34	1.178 ± 0.002bc	0.152	0.096	67.37	119.09	1.120 ± 0.020a	0.140	0.116
Mean	59.11	100.22	1.154 ± 0.014			59.81	106.17	1.114 ± 0.028		
**PI CONC. (μg/ml)**	***P. heterotricha***					***P. liguliformis***				
	**Mean PI fluorescence**		**Mean 2C-value (pg) ± SD**	**Mean RSE (%) sample**	**Mean RSE (%) standard**	**Mean PI fluorescence**		**Mean 2C-value (pg) ± SD**	**Mean RSE (%) sample**	**Mean RSE (%) standard**
	**Sample**	**Standard**				**Sample**	**Standard**			
10	54.68	37.16	1.336 ± 0.048a	0.198	0.234	64.90	40.13	1.460 ± 0.056a	0.124	0.183
25	72.83	49.43	1.330 ± 0.034a	0.189	0.185	68.86	41.72	1.488 ± 0.012a	0.118	0.155
50	64.14	42.65	1.356 ± 0.044a	0.171	0.171	75.46	44.85	1.518 ± 0.044a	0.114	0.154
100	70.21	47.33	1.338 ± 0.032a	0.164	0.163	81.23	47.88	1.532 ± 0.034a	0.105	0.157
150	59.29	38.65	1.384 ± 0.054a	0.171	0.154	90.03	52.50	1.546 ± 0.080a	0.113	0.148
200	75.39	51.35	1.324 ± 0.028a	0.165	0.144	85.18	50.92	1.510 ± 0.028a	0.108	0.133
Mean	66.14	44.43	1.344 ± 0.040			77.61	46.33	1.508 ± 0.036		

*Letters shared (within each species) indicated no significant differences in mean 2C-value (Tukey's HSD test P = 0.05). SD, Standard deviation. Each value is the mean of four replicates*.

### Effect of darkness and centrifugation

Our results demonstrate that incubation in absolute dark at 4°C did not improve the quality of the histograms (Figure S2). Moreover, poor-quality histograms were obtained after centrifugation (data not shown).

### Universality of the optimized methodology and new genome size estimates

Our comparative tests showed that FCM methodology using LB01 buffer supplemented with 100 μg ml^−1^ PI and staining for 40 min yielded the best results for genome size measurement in *Primulina*. We further tested performance of this optimal FCM protocol in other genera of Gesneriaceae, and found that it was suitable for *Hemiboea, Lysionotus, Briggsia*, and *Aeschynanthus*, with the values of CV falling below 5% (Table 4). The results revealed up to a 1.67-fold 2C DNA amount difference between the smallest (*H. gracilis*; 1.62 pg) and the largest (*L. pauciflorus*; 2.71 pg) genomes, indicating substantial interspecific genome size variation in Gesneriaceae (Table 4, Figure S3). Specifically, the genome size of *H. henryi* was determined based on analysis of 39 individuals from 17 populations across the entire geographical distribution, thereby representing the extent and pattern of intraspecific nuclear DNA content variation in this species. Significant differences in the DNA amount were detected among populations (*F* = 4.5, *P* = 0.001), where the mean 2C-value varied between 1.63 and 2.48 pg (Table 4, Figure S3). Intraspecific genome size variation was also apparent in *H. gracilis* and *L. pauciflorus*, the genome size of which differed by 5.10 and 11.0%, respectively.

**Table 4:** **Summary information of the nine species of Gesneriaceae included in this study**.

**Genus**	**Species**	**No. of pop**.	**Pop code**	**Pop locality**	**Coordinates**	**No. of indiv**.	**2C-value ± SD (pg)**	**Intraspecific variation (%)**	**CV (%)**
*Hemiboea*	*Hemiboea henryi*	17	HF02	Hefeng, Hubei	29.97°N/110.22°E	3	1.85 ± 0.01		4.66
			XE02	Xuanen, Hubei	29.70°N/109.57°E	1	1.98		4.87
			JH02	Jianghua, Hunan,	24.95°N/111.72°E	3	1.63 ± 0.02		4.70
			WG01	Wugang, Hunan	26.65°N/110.64°E	3	2.48 ± 0.02		3.55
			HNLW06	Linwu, Hunan	25.34°N/112.47°E	3	1.69 ± 0.03		4.86
			XN01	Xinning, Hunan	26.62°N/111.21°E	3	1.70 ± 0.01		4.75
			GZ01	Guzhang, Hunan	28.73°N/109.94°E	2	1.82 ± 0.03		4.51
			SZ06	Sangzhi, Hunan	29.67°N/109.82°E	4	1.70 ± 0.17		4.93
			HNLS07	Longshan, Hunan	29.27°N/109.38°E	3	2.40 ± 0.49		3.78
			SP05	Shuangpai, Hunan	26.07°N/111.93°E	1	1.91		4.81
			GZ04	Guzhang, Hunan	28.70°N/109.90°E	2	1.84 ± 0.01		4.39
			DX01	Daoxian, Hunan	25.50°N/111.47°E	3	1.71 ± 0.03		4.32
			FH02	Fenghuang, Hunan	28.11°N/109.50°E	1	1.95		4.62
			YS04	Yangshan, Guangdong	24.18°N/112.56°E	1	1.92		4.96
			JFS03	Nanchuan, Chongqing	29.05°N/107.13°E	3	1.81 ± 0.10		4.58
			GZKY05	Kaiyang, Guizhou	26.94°N/106.97°E	2	2.39 ± 0.60		4.42
			GZDY03	Duyun, Guizhou	26.35°N/107.48°E	1	2.09		3.37
			Overall			39	1.91 ± 0.33	52.1%	4.46
	*H. cavaleriei* var. *paucinervis*	1	GXNP01	Napo, Guangxi	23.31°N/105.62°E	1	2.10	–	4.44
	*H. wangiana*	1	GZXY02	Xingyi, Guizhou	25.01°N/104.91°E	1	2.02	–	4.15
	*H. gracilis*	2	HNLS07	Longshan, Hunan	29.27°N/109.38°E	2	1.65 ± 0.03		4.58
			SZ09	Sangzhi, Hunan	29.67°N/109.82°E	1	1.57		4.16
			Overall			3	1.62± 0.05	5.10	4.44
*Lysionotus*	*Lysionotus pauciflorus*	7	HNLS03	Longsha, Hunan	29.20°N/109.53°E	7	2.63 ± 0.12		3.40
			WG06	Wugang, Hunan	26.65°N/110.64°E	4	2.69 ± 0.17		3.59
			SP06	Shuangpai, Hunan	26.07°N/111.93°E	1	2.92		3.61
			GZLB03	Libo, Guizhou	25.42°N/107.86°E	2	2.72 ± 0.06		4.07
			GZDY04	Duxun, Guizhou	26.35°N/107.48°E	2	2.70 ± 0.00		3.63
			HNBT07	Baoting, Hainan	18.60°N/109.51°E	2	2.71 ± 0.17		3.18
			GXZY01	Ziyuan, Guangxi	26.13°N/110.69°E	4	2.84 ± 0.05		3.91
			Overall			21	2.71 ± 0.14	11.0	3.61
	*L. sangzhiensis*	1	SZ07	Sangzhi, Hunan	29.67°N/109.82°E	1	2.53	–	4.02
*Aeschynanthus*	*Aeschynanthus moningerae*	1	HNBT06	Baoting, Hainan	18.60°N/109.51°E	1	2.40	–	3.59
	*A. acuminatus*	1	GDSZ02	Shenzhen, Guangdong	22.57°N/114.21°E	1	2.10	–	4.29
*Briggsia*	*Briggsia mihieri*	1	GZSY05	Suiyang, Guizhou	28.23°N/107.29°E	1	2.02	–	4.06

*The table indicates the number of individuals and populations per species used for flow cytometry, the population locality, the coordinates, the 2C DNA content (mean 2C-value with standard deviation and intraspecific variation), and the CV (the average coefficient of variation)*.

*Each value is the mean of three replicates*.

## Discussion

### The presence of PI intercalation inhibitors in *Primulina*

Phytochemical compounds are reported to decrease the fluorescence intensity of PI-stained nuclei and consequently lead to inaccurate results (Greilhuber et al., 2007; Bennett et al., 2008). Our results confirmed that the existence of secondary metabolites influence the FCM estimations in most *Primulina* species except *P. subrhomboidea* (Table S1, Figure 2). The difference in the effects of inhibitors in various species can be attributed to their distinct composition and proportion of phytochemical compounds. For example, the level and activity of DNA PI staining inhibitors in *P. roseoalba, P. lunglinensi*, and *P. hedyotidea* were significantly lower than those in *P. liguliformis, P. huaijiensis, P. linearifolia*, and *P. heterotricha*. The karst environment, characterized by low soil-water content, periodic water deficiency and poor nutrients, might have exerted strongly selective stress on plant species. Previous studies documented that increased biosynthesis of secondary metabolites can be induced by nutritional deficiency and abiotic stress, such as drought, high light, and low temperature (Steyn et al., 2002). Thus, karst environment might trigger the biosynthesis of secondary metabolites in most *Primulina* species. It should be noted that approximately 300 chemical compounds have been reported in *Primulina* or other Gesneriaceae species, including flavonoids, terpenes, and steroids, phenolic glucosides, simple phenolics (Verdan and Stefanello, 2012). However, the chemical nature of the compounds that inhibit DNA staining in the analyzed *Primulina* species is yet to be determined.

### Significant effect of methodology on genome size estimations in *Primulina*

The present study confirmed that cytosolic compounds can affect genome size estimations in *Primulina*, highlighting the need for optimizing FCM methodology in the genus and other plants. Although the staining regimen showed a negligible effect on the value of genome size estimates in *Primulina*, both PI concentration and staining duration influenced the quality of the histograms. Incubation in absolute dark at 4°C and centrifugation did not improve the quality of the histograms. However, our results showed that the combined processing of target and standard species reduces potential variability and improves accuracy in determining genome size. Nevertheless, combined processing did not eliminate the effect of inhibitors on DNA content estimation, as evidenced by the variation produced by different buffers.

Buffer choice accounted for up to 6.83–21.4% of the estimated genome size differences in *Primulina* and also affected the quality and consistency of data. Buffer choice also seriously impacted FSC and SSC profiles (reflecting the size and granularity of nuclei, respectively), indicating a significant influence of buffers on the consistency of the examined nuclei structural properties. The significant influence of buffer choice can be explained by the ability of different buffers to counteract the detrimental effect of endogenous fluorescence inhibitors (Loureiro et al., 2006, 2007a,b). Consistent with the results in *Sinningia* (Gesneriaceae) (Zaitlin and Pierce, 2010), the CV-values obtained with the Partec CyStain PI Absolute P kit varied from 4.4 to 15.67% across species (data not shown). Only two (*P. lunglinensis* and *P. hedyotidea*) of the eight species exhibited CV-values below 5%, suggesting that the Partec CyStain PI Absolute P kit was not a good choice for genome size studies in most Gesneriaceae species. On the contrary, the Partec, Laat's, Galbraith's, and Tris-MgCl_2_ buffers, generally produced histograms with the poorest quality and genome size estimates with the highest variability in most species. Woody plant buffer produced good-quality histograms but the obtained genome size appeared to be very different from the mean value and had a higher SE. Our results showed that LB01 was the best buffer for genome size estimation in *Primulina*, producing the best-quality histograms and consistent genome size estimates. LB01 contains β-mercaptoethanol, an antioxidant that could bind free radicals. Similarly, buffer MgSO_4_ generated high-quality histograms, which may be due to the presence of dithiothreitol (DTT), a typical reducing agent. The reducing function of these compounds probably counteracts the effect of endogenous fluorescence inhibitors, such as tannins or other phenolic compounds detected in *Primulina* (Verdan and Stefanello, 2012).

### High methodology universality and genome size variation reports for gesneriaceae

The low CV-values (< 5%) commonly detected in the four other genera of Gesneriaceae indicate the optimized methodology is highly universal for *Primulina*. These results will be helpful for genome size studies in this family. In fact, the optimized methodology has been used to obtain genome size of 101 *Primulina* species (Kang et al., 2014). We also report the genome size for nine species from the genera *Hemiboea, Lysionotus, Briggsia*, and *Aeschynanthus*, for the first time. The number of species and individuals analyzed for each genus depended on the sample availability. Similar to our findings in *Primulina* (2C = 1.12–2.54 pg), the genome sizes detected in these genera (2C = 1.62–2.71 pg) also fall into the very small category (1C = 1.4 pg), as defined by Soltis et al. (2003) and Leitch et al. (2005). Our results also indicate a substantial genome size variation both within and among species. Specifically, we found high intraspecific genome size variation (52.1%) in *H. henryi*. Given the extensive sampling across the species distribution range and the low CV-values obtained in our study, our estimates probably reflect real genome size variation among populations of *H. henryi*. Although genome size is considered more likely to be constant at the species level, intraspecific variation is increasingly detected in plant species (e.g., Duchoslav et al., 2013; Huang et al., 2013). Similarly, our studies in *Primulina* revealed as much as 41.23% intraspecific variation in *P. linearifolia* (Kang et al., 2014). Significant variations in genome size can be attributed to several sources, including variation in chromosome number or ploidy level or the amount of repetitive and non-coding DNA, which is considered the major mechanism responsible for changes in genome size (Bennetzen et al., 2005). Moreover, genome size variation could be a result of local adaptation along ecogeographic gradients (Kang et al., 2014; Jordan et al., 2015). *H. henryi* is one of the most widely distributed species of Gesneriaceae in China, which occurs in diverse habitats, including limestone karst and acidic soils. The complexity of selection patterns and variation in adaptation to environments among lineages may generate population-specific relationships; thus, the present study highlights the necessity for further research into detailed ecological or geographical factors to elucidate the evolutionary patterns of genome size in *H. henryi*. Nevertheless, considering the high intraspecific genome size variation observed in *H. henryi*, we suggest further taxonomic treatment for this species based on both molecular and morphological data in the future.

## Conclusions

This study quantitatively evaluated FCM methodology effects on genome size estimation in the genus *Primulina*. Our results confirmed that the existence of secondary metabolites can affect genome size determination and highlighted the necessity of optimizing the FCM methodology prior to obtaining a reliable genome size for a given taxon.

## Author contributions

This study was conceived by MK and JW. Collection and identification of field material was performed by MK. Sample preparation, nuclei isolation, and flow cytometry analyses were performed by JW and JL. Data analysis was conducted by JW. JW and MK wrote the paper. All authors read and approved the final manuscript.

### Conflict of interest statement

The authors declare that the research was conducted in the absence of any commercial or financial relationships that could be construed as a potential conflict of interest.

## Abbreviations

CV: coefficient of variation
FCM: flow cytometry
PI: propidium iodide.
