# Supplementary material for: Quantitative testing of the methodology for genome size estimation in plants using flow cytometry: a case study of the Primulina genus
Source: Front Plant Sci. 2015 May 19;6:354. doi: 10.3389/fpls.2015.00354 (PMC4436564; doi:10.3389/fpls.2015.00354)
Supplement: Supplementary file 1 [file Table1.DOC]

Table S1. Propidium iodide (PI) fluorescence of nuclei from simultaneously processed (Standard 1) standard (*S. lycopersicum* or *O. sativa ssp. japonica*) and independently-chopped (Standard 2) using Partec CyStain PI Absolute P kit.

| Species | Standard | Mean PI fluorescence | | Standard 1 /Standard 2 ratio |
| --- | --- | --- | --- | --- |
|  |  | Standard 1 | Standard 2 |  |
| *Primulina linearifolia* | *Solanum lycopersicum* | 94.74 | 113.50 | 0.84 |
| *P. huaijiensis* | *S. lycopersicum* | 91.18 | 113.50 | 0.80 |
| *P. heterotricha* | *Oryza sativa ssp. japonica* | 35.49 | 41.27 | 0.86 |
| *P. liguliformis* | *O. sativa ssp. japonica* | 30.17 | 41.27 | 0.73 |
| *P. roseoalba* | *O. sativa ssp. japonica* | 41.05 | 41.27 | 0.99 |
| *P. lunglinensis* | *O. sativa ssp. japonica* | 39.65 | 41.27 | 0.96 |
| *P. hedyotidea* | *O. sativa ssp. japonica* | 39.31 | 41.27 | 0.95 |
| *P. subrhomboidea* | *O. sativa ssp. japonica* | 41.24 | 41.27 | 1.00 |

Each value is the mean of three replicates.

Table S2. CV-value of eight *Primulina* species obtained with various buffers.

| Buffer | *P. linearifolia* | *P. huaijiensis* | *P. heterotricha* | *P. liguliformis* | *P. roseoalba* | *P. lunglinensis* | *P. hedyotidea* | *P. subrhomboidea* |
| --- | --- | --- | --- | --- | --- | --- | --- | --- |
| MgSO_4_ | 4.46 | 7.28 | 4.20 | 5.29 | 4.39 | 4.46 | 4.71 | 4.95 |
| Partec | 5.81 | 15.67 | 6.82 | 7.03 | 5.28 | 4.37 | 4.74 | 5.67 |
| de Laat’s | 5.67 | 9.63 | 5.70 | 5.30 | 5.60 | 4.47 | 6.82 | 6.43 |
| Galbraith’s | 11.19 | 9.99 | 5.90 | 7.74 | 6.10 | 4.83 | 4.69 | 6.60 |
| LB01 | 4.45 | 4.97 | 4.69 | 4.69 | 4.32 | 2.01 | 4.11 | 4.07 |
| Tris.MgCl_2_ | 7.11 | 8.59 | 6.21 | 6.41 | 5.35 | 5.84 | 7.83 | 5.25 |
| General purpose | 6.86 | 9.02 | 6.32 | 6.57 | 5.40 | 4.07 | 5.06 | 4.83 |
| Woody Plant | 4.58 | 13.56 | 7.28 | 4.72 | 5.65 | 4.07 | 5.40 | 5.22 |

Each value is the mean of four replicates.

Fig. S1. Ungated histograms and scattergram outputs for *Primulina linearifolia* using LB01 buffer (A) and de Laat’s buffer (B).


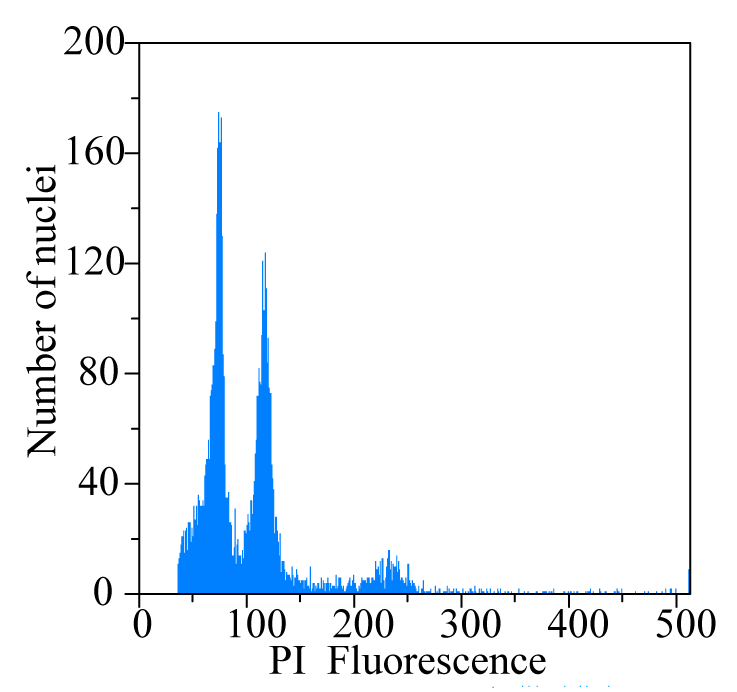

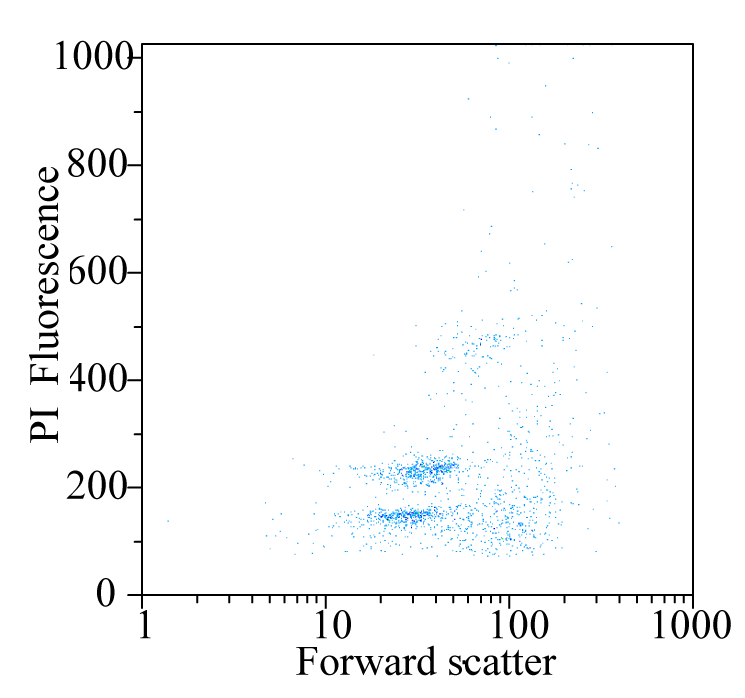

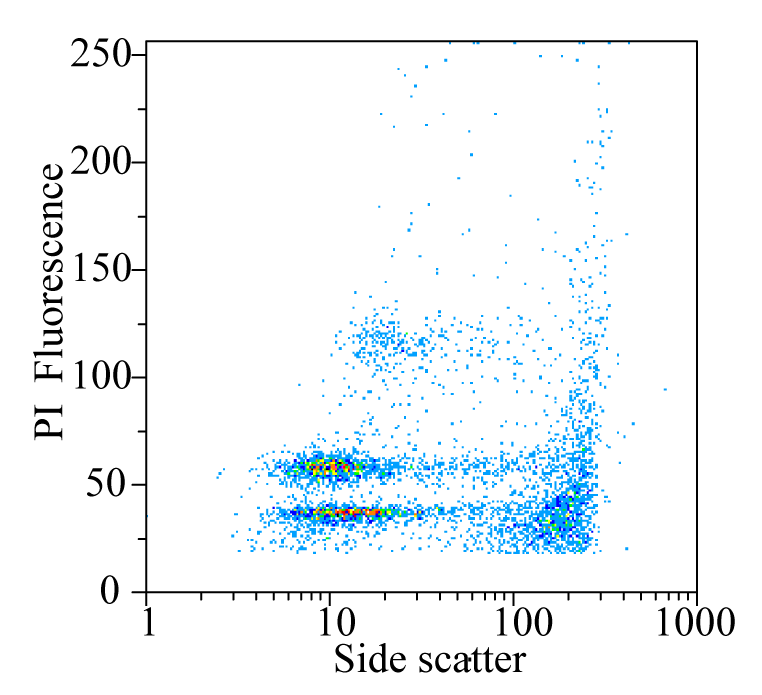


A


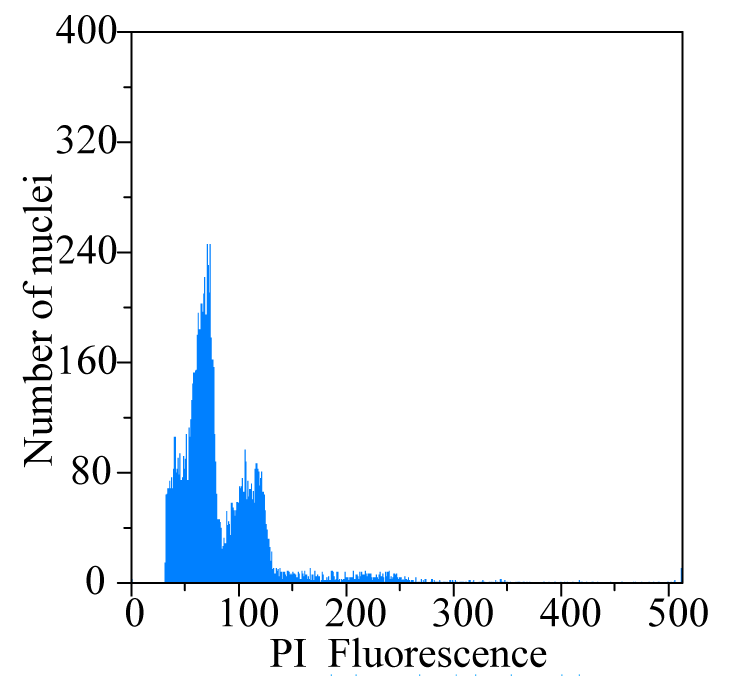

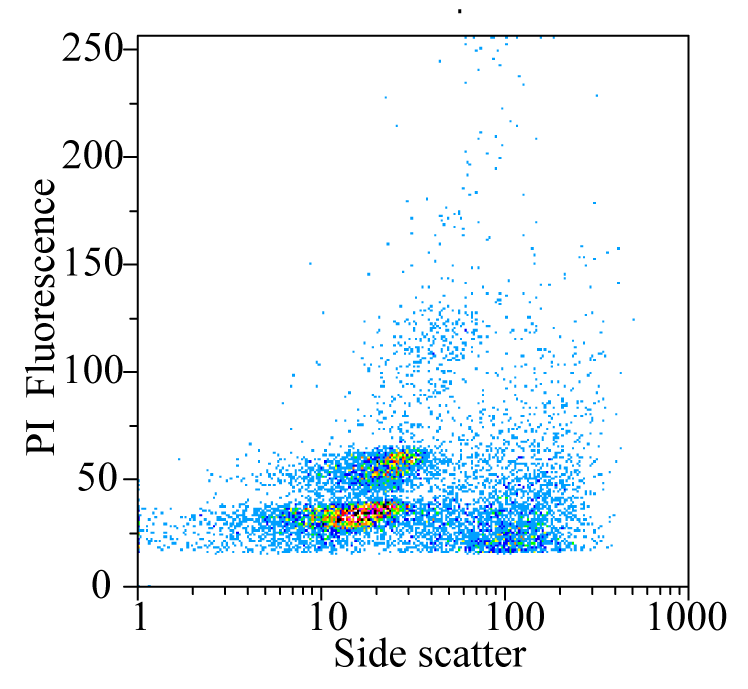

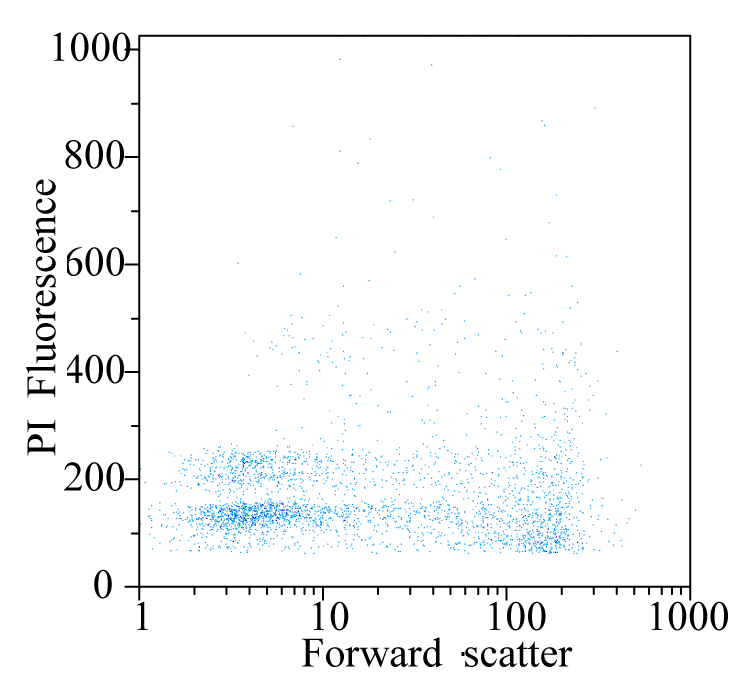


B

Fig. S2. Representative ungated histograms of *Primulina* with treatment of one-day period in the dark at 4 °C.


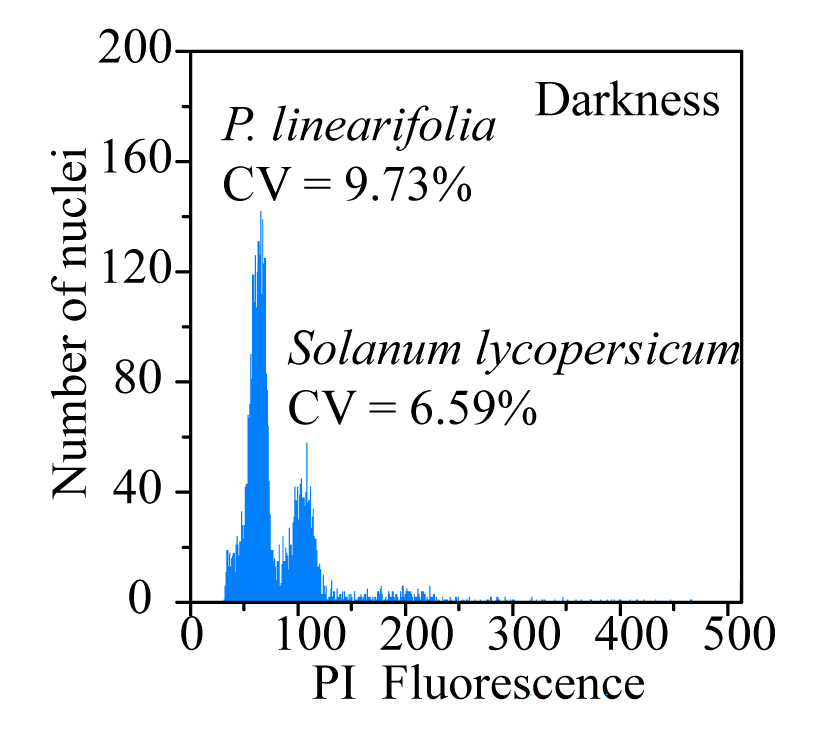

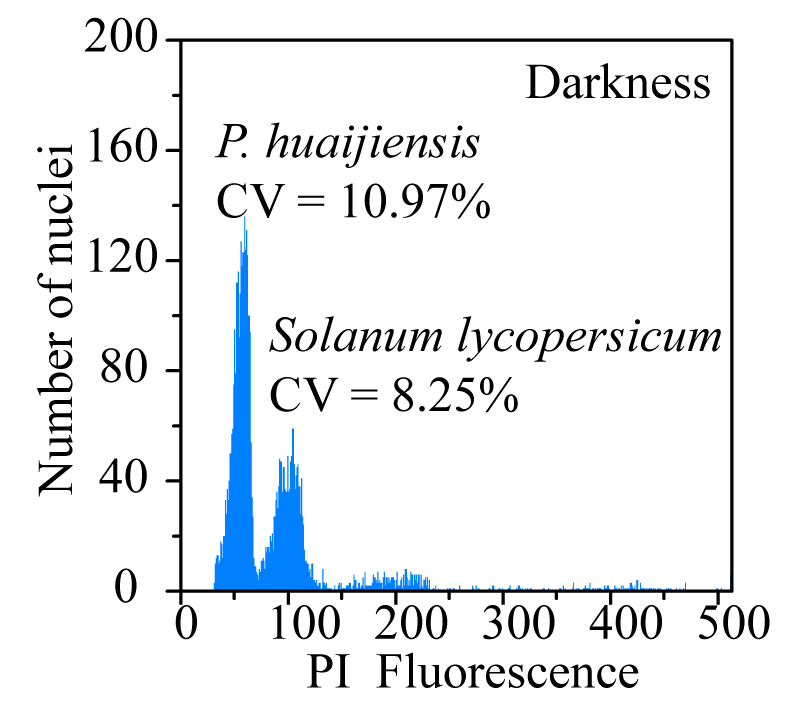


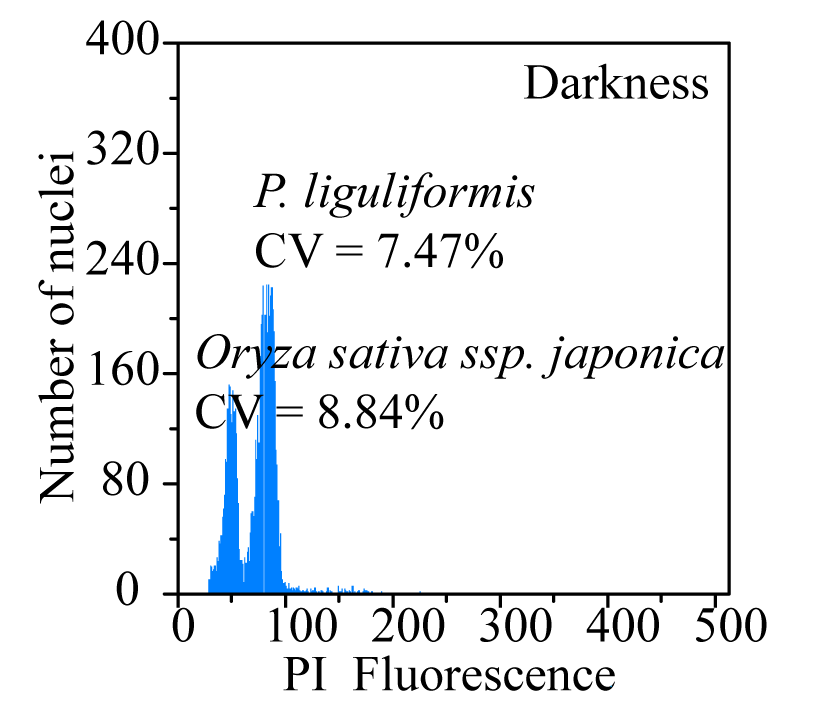

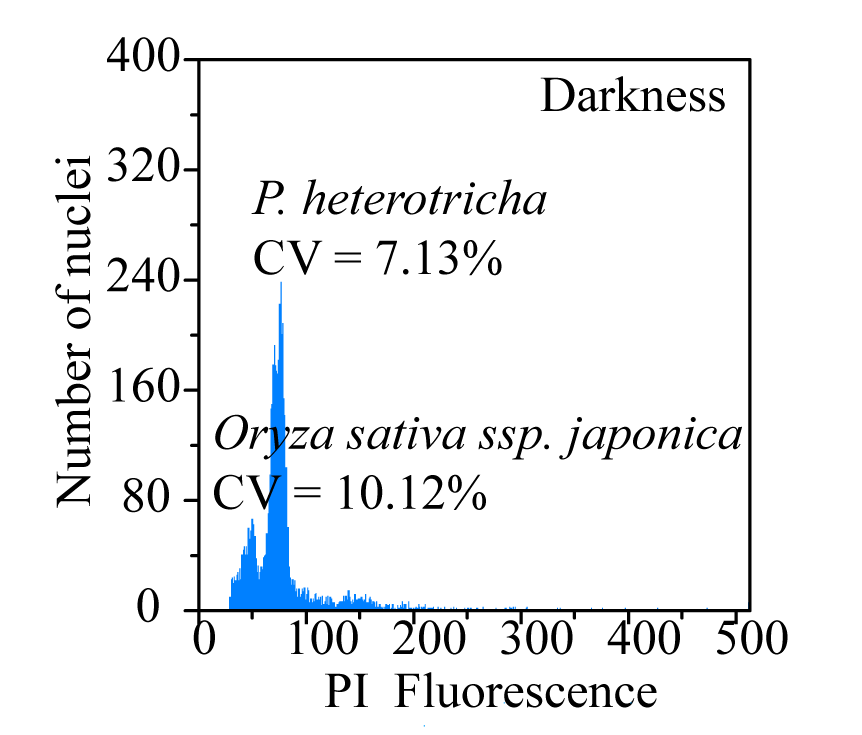


Figure S3. Histograms showing the inter-specific (A, *Hemiboea henryi* vs. *Lysionotus pauciflorus*) and intra-specific (B, *H. henryi)* genome size variation in Gesneriaceae.


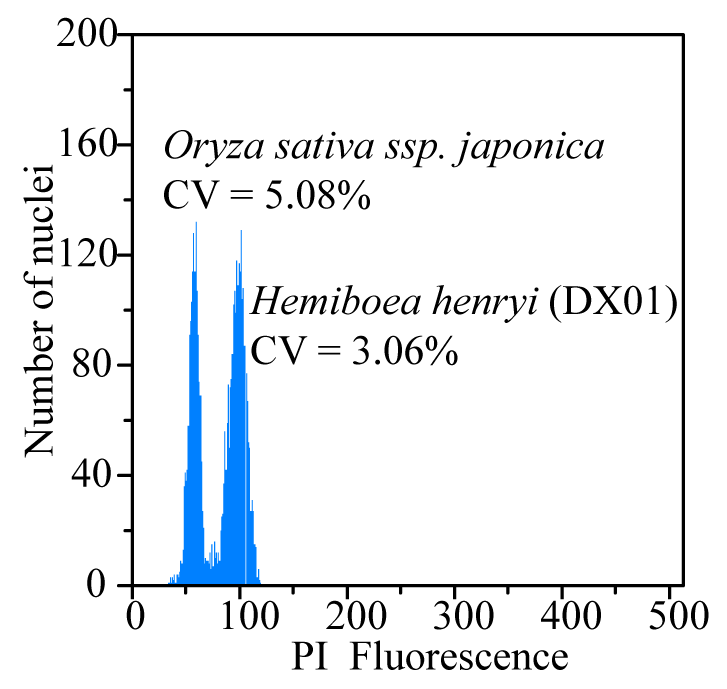

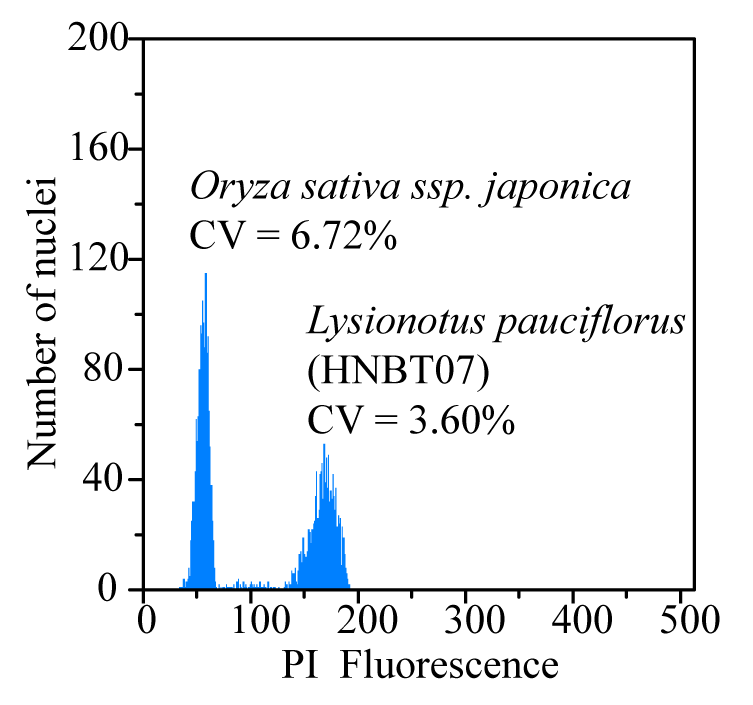

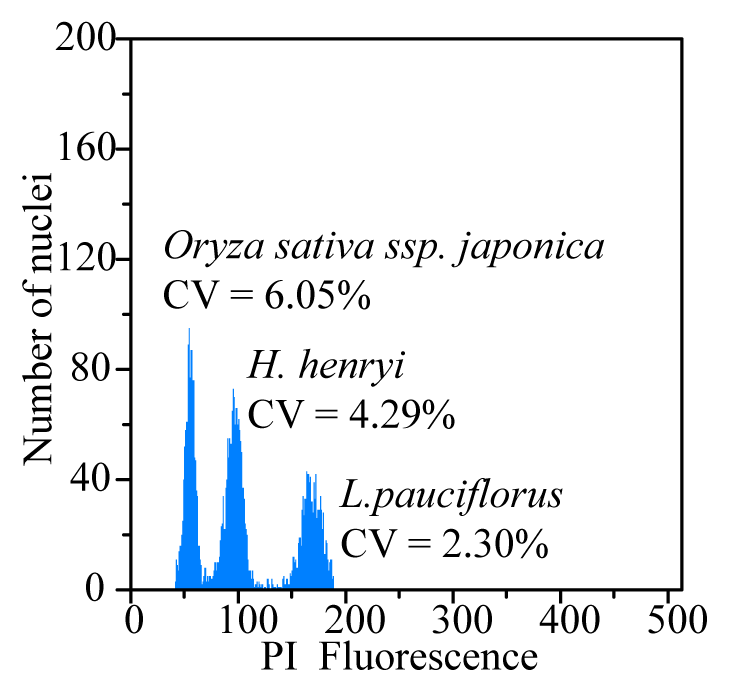


A


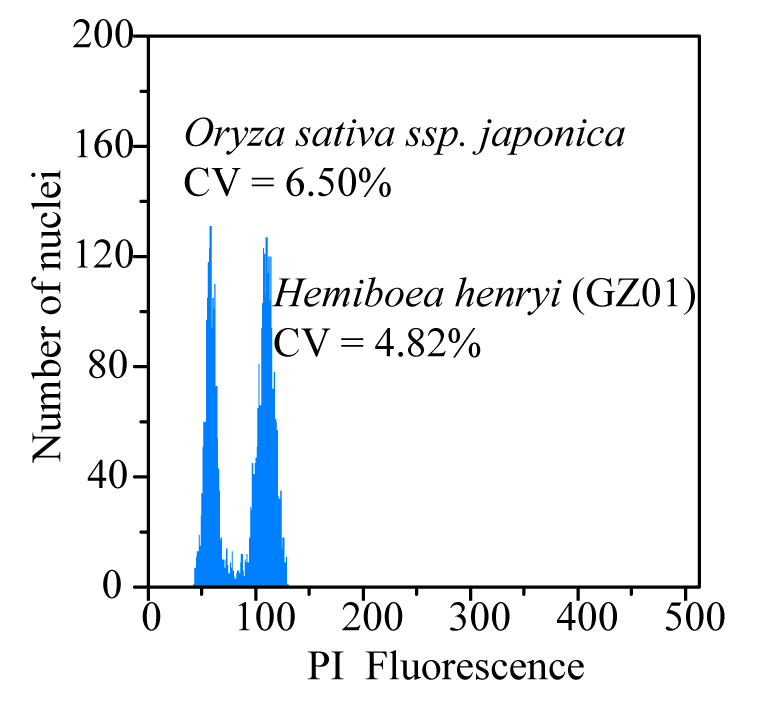

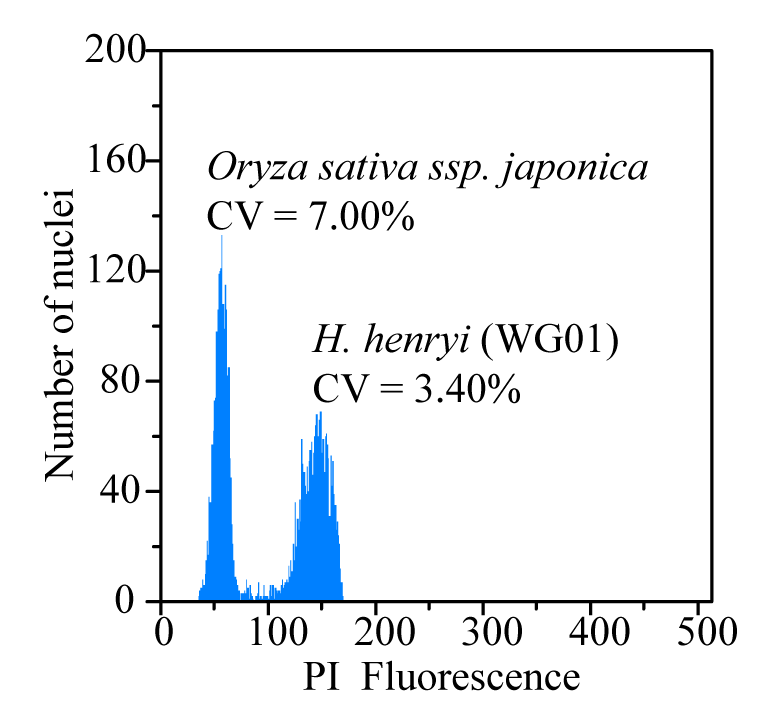

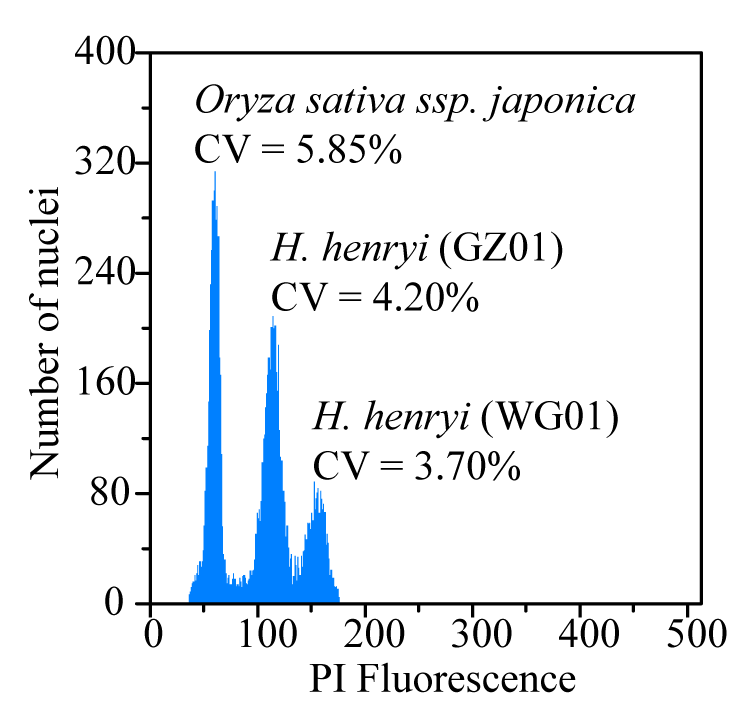


B
